# Doubling the Dose of Bevacizumab Beyond Progression in Metastatic Colorectal Cancer–the Experience of a Tertiary Cancer Center

**DOI:** 10.3389/fphar.2021.487316

**Published:** 2021-03-11

**Authors:** Călin Căinap, Ovidiu-Vasile Bochiş, Cătălin Vlad, Raluca Popita, Patriciu Achimaş-Cadariu, Andrei Havasi, Andreea Vidrean, Alexandra Dranca, Andra Piciu, Anne-Marie Constantin, Tiberiu Tat, Maniu Dana, Ovidiu Crişan, Cosmin Vasile Cioban, Ovidiu Bălăcescu, Ovidiu Coza, Loredana Bălăcescu, Monica Mihaela Marta, Madalina Bota, Simona Căinap

**Affiliations:** ^1^ “Prof Dr Ion Chiricuta” Institute of Oncology, Cluj-Napoca, Romania; ^2^ Department of Oncology, “Iuliu Hatieganu” University of Medicine and Pharmacy, Cluj-Napoca, Romania; ^3^ Department of Surgical Specialities, “Iuliu Hatieganu” University of Medicine and Pharmacy, Cluj-Napoca, Romania; ^4^ Department of Morphological Sciences, “Iuliu Haţieganu” University of Medicine and Pharmacy, Cluj-Napoca, Romania; ^5^ Department of Anesthesia and Intensive Care I, “Iuliu Hatieganu” University of Medicine and Pharmacy, Cluj-Napoca, Romania; ^6^ Faculty of Physics, Babes-Bolyai University, Cluj-Napoca, Romania; ^7^ Faculty of Pharmacy, “Iuliu Hatieganu” University of Medicine and Pharmacy, Cluj-Napoca, Romania; ^8^ Faculty of Dental Medicine, “Iuliu Hatieganu” University of Medicine and Pharmacy, Cluj-Napoca, Romania; ^9^ Department of Medical Education, “Iuliu Hatieganu” University of Medicine and Pharmacy, Cluj-Napoca, Romania; ^10^ Department of Mother and Child, “Iuliu Hatieganu” University of Medicine and Pharmacy, Cluj-Napoca, Romania

**Keywords:** colorectal, cancer, progression, bevacizumab, metastasis, double dose

## Abstract

**Background:** Colorectal cancer (CRC) is the third most common cancer in Europe, with an annual increase in incidence ranging between 0.4 and 3.6% in various countries. Although the development of CRC was extensively studied, limited number of new therapies were developed in the last few years. Bevacizumab is frequently used as first- and second-line therapy for management of metastatic CRC (mCRC). The aim of this study is to present our experience with using bevacizumab beyond disease progression at different dosage levels in mCRC patients, in terms of overall survival, progression-free survival, time to treatment failure, and toxicities.

**Methods:** We performed a consecutive retrospective analysis of patients with confirmed mCRC who were treated with bevacizumab at "Prof Dr. Ion Chiricuta" Institute of Oncology, Cluj-Napoca, Romania. We included patients who had received bevacizumab as first- or second-line therapy and further stratified them according to the dose administered as a second-line (either standard dose of 5 mg/kg every 2 weeks or 7.5 mg/kg every 3 weeks, or double dose of 10 mg/kg every 2 weeks or 15 mg/kg every 3 weeks–depending on the classical chemotherapy partner). All patients had received bevacizumab beyond progression (BYP) which is defined as continuing bevacizumab administration through second-line treatment despite disease progression. In each group, we evaluated the prognostic factors that influenced survival and treatment outcome.

**Results:** One hundred and fifty-one (151) patients were included in the study. Themedian age of patients receiving double dose bevacizumab (DDB) and standard dose bevacizumab (SDB) was 58 years (range 41–71) and 57 years (range 19–75), respectively. The median overall survival in the DDB group was 41 months (range 27–49) compared to 25 months (range 23–29) in the SDB group (*p* = 0.01 log-rank test). First-line oxaliplatin-based treatment was used more frequently regardless of group, while irinotecan-based more frequently used as a second-line treatment (*p* = 0.014). Both oxaliplatin- and irinotecan-based regimens were found to be suitable partners for BYP. Statistical analysis revealed that dose intensity, primary tumor location, and cumulative exposure to BYP had significant influence on survival.

**Conclusion:** Doubling the dose of bevacizumab after first progression may improve survival in mCRC patients. Increasing bevacizumab dose intensity could override the prognostic impact of primary tumor location in patients receiving double the dose of bevacizumab after first disease progression.

## Introduction

According to the latestdata released by GLOBOCAN in 2018, colorectal cancer (CRC) is one of the most common types of cancer worldwide, being the third most frequent and the second most fatal malignancy (Ferlay et al., 2018). In Europe, CRC is the third most common cancer, with the highest incidence rates registered in countries such as Hungary, Slovenia, Slovakia, Norway, and the Netherlands (Ferlay et al., 2018). In Romania, CRC is the second most frequent malignancy after lung cancer in both genders, with a rapidly increasing incidence (Ferlay et al., 2018). The annual increase in incidence in the different European countries ranges from 0.4 to 3.6%.According to the latest reports, the age of disease onset appears to be decreasing. Vuik et al. analyzed the incidence of CRC in the last 25 years in Europe, and revealed an increase in the incidence of the disease among adults aged 20–49 years of age, compared with initial data which showed a predisposition for CRC starting with fifth decade (Vuik et al., 2019).

Since CRC treatment can be curative in the localized and locoregional disease, early diagnosis through national screening programs is essential. However, up to 44% of patients with loco regional disease will develop metastases despite treatment (Bray et al., 2018). In such relapsed cases, as well as in the 20% of CRC patients presenting with metastasis at diagnosis (Edwards et al., 2014), overall survival (OS) can exceed 30 months in fit patients who benefit fromthe triple-agent chemotherapy regimen (FOLFOXIRI) combined with targeted therapy (Qiu et al., 2015).

Despite the discoveries made in the last few years and the research conducted in order to highlight the mechanisms of CRC pathogenesis, the processes that allow cancer cells to migrate, invade and metastasize to other parts of the body have not yet been fully described (Esin and Yalcin, 2016; Coyle et al., 2017).

Although genomic instability—microsatellite instability (MSI), chromosomal instability (CIN), and CpG island methylator phenotype (CIMP) are known to contribute to the development of CRC (Hong, 2018), a limited number of new therapies for metastatic CRC (mCRC) patients have been developed in the last few years. Therapeutic options currently available to treat mCRC include the classical chemotherapy backbone–fluoropyrimidine with oxaliplatin or irinotecan, combined with either an anti-angiogenic agent, or anti-epidermal growth factor receptor (EGFR) antibodies. There are several classesof drugs which target malignant angiogenesis, such as anti-vascular endothelial growth factor (VEGF) antibodies, proteins with binding portions for the extracellular domains of human VEGF receptors 1 and 2, which will retain tumor-released VEGF (a VEGF trap: aflibercept, for example), or protein kinase inhibitors which target angiogenic, stromal and oncogenic receptor tyrosine kinase (RTK) (Stivarga, 2020; Zaltrap, 2020). While anti-EGFR antibodies are used exclusively for mCRC patients with wild-type RAS, anti-angiogenic drugs can be of benefit in all patients regardless of RAS status.Bevacizumab (anti-VEGF antibody) is frequently used as first-orsecond-line therapy for the management of mCRC. Beyond the first progression of the disease, standard dose bevacizumab (SDB) or double dose bevacizumab (DDB) can be administered (Avastin, 2020). New target drugs have been approved for use in patients with mCRC such as immunotherapy like pembrolizumab–approved in MSI-high mCRC patientsas well asunresectable or metastatic solid tumors with MSI-H ordeficient mismatch repair (dMMR). BRAF inhibitors–dabrafenib and MEK inhibitors for mCRC BRAF mutant are still under investigations (Al-Husein et al., 2012; Cutsem et al., 2016; Kuramochi et al., 2017; Keytruda, 2020). Human epidermal growth factor receptor (HER2) amplification seems to be a valuable new target in mCRC. Despite its prevalence of 2% in the general population of mCRC patients, it seems to be linked to primary resistance to anti-EGFR agents (Dienstmann et al., 2018). Although phase III trials are not available, the response rate to anti-HER2 agents reached 38% in the MyPathway study (Dienstmann et al., 2018).

The aim of this study is to present our experience with using bevacizumab beyond disease progression at different dosage levels in mCRC patients, in terms of OS, progression-free survival (PFS), time to treatment failure (TTF), and toxicities.

## Materials and Methods

### Study Population

This study was conducted to generate data from a tertiary care center of excellence in the treatment of mCRC in Romania. We present the treatment strategies, prognostic factors, and survival data of them CRC patients treated between 2009 and 2017 outside of a clinical trial. This study retrospectively included mCRC patients who used bevacizumab as first-line and second-line treatment. Two treatment options are used in our cancer center in which the first is to continue SDB and the other is to consider DDB through second-line treatment. Hence, mCRC patients in this study were classified according to the dose of bevacizumab administered beyond progression. Bevacizumab beyond progression (BYP) was defined as the continuation of bevacizumab treatment in the second-line of systemic therapy despite disease progression proven through imaging techniques. SDB implies that patients continued the same dose of bevacizumab (5 mg/kg every 2 weeks or 7.5 mg/kg every 3 weeks) administered as first-line treatment, while DDB indicates doubling the dose of bevacizumab (10 mg/kg/every 2 weeks or 15 mg/kg every 3 weeks). The decision to maintain SDB or switch to DDB through the second-line treatment was made by oncologists based on bevacizumab toxicity and tolerance through the first-line treatment phase. The study was approved by the Ethics Committee of “Prof.Dr. Ion Chiricuta” Institute of Oncology,Cluj-Napoca,Romania through decision No 42/8 December 2015.

***The inclusion criteria*** were: age of 18 years or older, histologically confirmed diagnosis of CRC, lab tests adequate for chemotherapy and no medical contraindication to chemotherapy, at least one metastatic site, Eastern Cooperative Oncology Group (ECOG) performance status of 0–2, bevacizumab administration in first and second-line treatment, adequate follow-up (at least monthly clinical checkup and CT-scan every 3–4 months).

***The exclusion criteria*** were: previous administration of chemotherapy for the metastatic stage, uncontrolled comorbidities, poor performance status (ECOG ≥3), inadequate labtests, hypersensitivity to the active substance, heart failure (NYHA grade >2), uncontrolled hypertension, acute myocardial infarction (within 6 months prior to start chemotherapy) and pregnancy.

According to the literature, the benefit of systemic treatment in mCRC is controversial especially in patients with poor ECOG performance status, with no survival advantage over the best supportive care (Crosara Teixeira et al., 2015). Therefore, patients with poor performance status were excluded from this study.

### Chemotherapy Regimens and Follow-up

The chemotherapeutic regimens used in this study were consistent with international guidelines: capecitabine-based (CAPEOX/XELOX or CAPIRI/XELIRI) 3 weeks regimen or 5-fluorouracil-based (FOLFOX4 or FOLFIRI) 2 weeks regimen, at the dosages displayed in Table 1. Dose modifications during treatment were allowed according to guideline recommendations (Cutsem et al., 2016; Messersmith, 2019).

**TABLE 1:** Chemotherapy used in combination with bevacizumab in first and second-line therapy.

Type of chemotherapy	Drug	Dose	Cycle length
FOLFOX4	5-FU	400 mg/m^2^ bolus in 15 min day 1 + 2	14 days
	5-FU	600 mg/m^2^ continuous perfusion over 22 h day 1 + 2	
	Leucovorin	400 mg/m^2^ in 2 h day 1 + 2	
	Oxaliplatin	85 mg/m^2^ in 2 h day 1	
FOLFIRI	5-FU	400 mg/m^2^ bolus in 15 min day 1 + 2	14 days
	5-FU	600 mg/m^2^ continuous perfusion over 22 h day 1 + 2	
	Leucovorin	400 mg/m^2^ in 2 h day 1 + 2	
	Irinotecan	180 mg/m^2^ day 1	
CAPEOX	Capecitabine	2,000 mg/m^2^ days 1–14	21 days
	Oxaliplatin	130 mg/m^2^ in 2 h day 1	
CAPIRI	Capecitabine	2,000 mg/m^2^ days 1–14	21 days
	Irinotecan	240 mg/m^2^ day 1	
Bevacizumab standard dose (SDB)	Bevacizumab + FOLFOX4 or FOLFIRI	5 mg/kg	Every 2 weeks
Bevacizumab standard dose (SDB)	Bevacizumab + CAPEOX or CAPIRI	7.5 mg/kg	Every 3 weeks
Bevacizumab double dose (DDB)	Bevacizumab + FOLFOX4 or FOLFIRI	10 mg/kg	Every 2 weeks
Bevacizumab double dose (DDB)	Bevacizumab + CAPEOX or CAPIRI	15 mg/kg	Every 3 weeks

5FU, 5 fluorouracil; m^2^-square meter; mg-milligram, kg-kilogram.

After first-line chemotherapy, most patients underwent maintenance therapy with a reduction in chemotherapy intensity until disease progression or surgical resection. The same standard or double dose bevacizumab was continued beyond disease progression in combination with a different chemotherapy partner. All patients were assessed by CT scan according to RECIST 1.1 (Eisenhauer et al., 2009).

### Statistical Analysis

For statistical analysis purposes, we defined overall survival (OS) as the period of time between the first cycle of chemotherapy and death, time to treatment failure (TTF) as the period of time between the first and the last cycle of bevacizumab chemotherapy, progression-free survival after first-line therapy (PFS1) as the time between the first and the last cycle of first-line chemotherapy and progression-free survival during second-line therapy (PFS2) as the time between the first and the last cycle of second-line chemotherapy. These definitions were similar to those in the Simkens CAIRO3 trial with TTF corresponding to the time to second progression (Simkens et al., 2015). We also defined PFS during second-line of therapy in the DDB group.

The main characteristics of the studied population were analyzed using Microsoft Excel 2010, followed by Chi-square test for association. For data reported as mean ± SD, *p*-values were calculated with *t*-test. Survival analyses were performed using R version 3.5.1 [R Core Team (2018). R: A language and environment for statistical computing. R Foundation for Statistical Computing, Vienna, Austria. URLhttp://www.R-project.org/.] in multiple steps. First, we used the Kaplan-Meier method to obtain survival curves. Second, we compared them using the log-rank test. Finally, we used Cox regression to generate Hazard Ratios (HRs) and corresponding 95% Confidence Intervals (CIs). The calculated *p*-values were two-sided, and *p*-values less than 0.05 were considered statistically significant. The Pearson correlation coefficients as well as the corresponding *p*-values were determined using Pearson correlation test, in Microsoft Excel 2010.

## Results

### Patient Characteristics

In a retrospective analysis of mCRC patients treated with chemotherapy in our institute between 2009 and 2017, we identified 162 patients who met the main inclusion criteria. Out of these, 151 met the criteria for “beyond progression”, while 11 were treated with bevacizumab through multiple lines or beyond the third-line of chemotherapy and therefore were excluded from the analysis.

First-line oxaliplatin-based chemotherapy was more frequently used than irinotecan-based therapy in the SDB group compared with the DDB group (*p* < 0.001), while irinotecan was more frequently used in the second-line after disease progression (*p* = 0.014) (Table 2). The median age was 57 years (range 19–75) in the SDB group and 58 in the DDB group (range 41–71), while approximately 80% of patients were under the age of 65 in both groups. The primary tumor was in the right side of the colon in 76.8% of cases. The liver, peritoneum and lungs were the most common metastatic sites in both the SDB and DDB groups. About 27.2% of patients had more than one metastatic site. No significant differences were noted between SDB and DDB patients regarding site and number of metastases. More male patients had left-sided cancer compared to female patients. Mean body mass index (BMI) was significantly higher in the DDB group compared to patients receiving SDB (*p* = 0.01). Regarding the RAS status, a reasonable proportion of patients did not have data; however no significant differences were observed between the SDB and DDB groups.

**TABLE 2:** Baseline characteristics of mCRC patients in SDB (*N* = 111) and DDB (*N* = 40) groups.

		SDB	DDB	*p*-value
Age **(**years)	Under 65	88 (79.3%)	35 (87.5%)	0.25
	Over 65	23 (20.7%)	5 (12.5%)	
Gender	Male	62 (58.9%)	24 (60.0%)	0.65
	Female	49 (44.1%)	16 (40.0%)	
BMI*(kg/m^2^)		25.17 ± 4.47 (14.19–36.51)	27.73 ± 5.03 (19.59–42.24)	**0.01**
Primary tumor	Left-sided	35 (23.17)		0.25
	Right-sided	116 (76.82%)		
Metastasis site	Liver	91 (58.0%)	30 (62.5%)	0.98
	Lung	19 (12.1%)	6 (12.5%)	
	Peritoneum	25 (15.9%)	6 (12.5%)	
	Lymph nodes	6 (3.8%)	2 (4.2%)	
	Bone	6 (3.8%)	1 (2.1%)	
	Other**	10 (6.4%)	3 (6.3%)	
Metastasis–organs involved	1	78 (70.3%)	32 (80.0%)	0.24
	>1	33 (29.7%)	8 (20.0%)	
First-line chemotherapy	Oxaliplatin-based	81 (73.0%)	9 (22.5%)	**<0.01**
	Irinotecan-based	30 (27.0%)	30 (75.0%)	
Second-line chemotherapy	Oxaliplatin -based	33 (29.7%)	20 (50%)	**0.01**
	Irinotecan-based	78 (70.3%)	19 (47.5%)	
RAS	Mutant	11 (9.9%)	6 (15%)	0.06
	Wild type	32 (28.8%)	5 (12.5%)	
	Not available	68 (61.3%)	29 (72.5%)	
T Stage	1	0 (0%)	1 (2.5%)	**<0.01**
	2	3 (2.7%)	1 (2.5%)	
	3	58 (52.3%)	27 (67.5%)	
	4	34 (30.6)	7 (17.5%)	
	Not available	16 (14.4%)	4 (10%)	
N stage	0	8 (7.2%)	6 (15.0%)	0.39
	1	29 (26.1%)	13 (32.5%)	
	2	48 (43.2%)	16 (40.0%)	
	Not available	26 (23.4%)	5 (12.5%)	
M Stage	0***	35 (31.5%)	14 (35%)	0.72
	1	72 (64.9%)	25 (62.5%)	
	Not available	4 (2.5%)	1 (2.5%)	

SDB: standard dose bevacizumab, DDB: double dose bevacizumab; BMI: body mass index; data are n (%); *p*-value was calculated with Chi-test.

*data are mean ± SD (range) and *p*-value was calculated with *t*-test; all tests were conducted at 0.05 level of significance. Bold the significant *p* value when we have multiple values.

**subcutaneous, ovaries.***M0–stage at initial diagnostic.

The median values of OS, TTF, PFS1, and PFS2 were significantly higher in the DDB group compared with the SDB group (Table 3).

**TABLE 3:** Median of OS, PFS, and TTF based on bevacizumab dose.

Endpoint	SDB	DDB	*p*-value
OS	25 (23–29)	41 (27–49)	**0.01**
TTF	19 (16–22)	24 (21–35)	**0.01**
PFS1	12 (11–14)	17 (15–22)	**0.01**
PFS2	5 (4–6)	9 (6–12)	**0.03**

SDB: standard dose bevacizumab, DDB: double dose bevacizumab, OS: overall survival, TTF: time to treatment failure, PFS1: progression-free survival during first-line treatment, PFS2: progression-free survival during second-line treatment; data are median (interquartile range) in months; p-values were calculated with log-rank test at 0.05 level of significance. p value statistical significant.

### Bevacizumab Toxicity

Regarding toxicity of bevacizumab, hypertension and proteinuria were more frequent in the DDB compared to the SD Bgroup. Of those, only proteinuria and hypertension grade 3 reached statistical significance (Table 4). These differences did not lead to significant treatment delays between the SDB and DDB groups.

**TABLE 4:** Bevacizumab toxicity in SDB and DDB groups.

Type of toxicity		DDB–number of patients (% from total number-40)	SDB- number of patients (% from total number-111)	*p*-value
Proteinuria	Grade 1	7 (17.5%)	10 (9%)	0.69
	Grade 2	4 (10%)	3 (2.7%)	
	Grade 3	3 (7.5%)	5 (4.5%)	
	Any grade	14 (35.0%)	18 (16.2%)	**0.01**
Hypertension	Grade 2	1 (2.5%)	10 (9%)	**0.04**
	Grade 3	7 (17.5%)	8 (7.2%)	
	Any grade	8 (20%)	18 (16.2%)	0.29
Thromboembolic events	Grade 2	1 (2.5%)	3 (2.7%)	0.17
	Grade 3	1 (2.5%)	0 (0%)	
	Any grade	2 (5%)	3 (2.7%)	0.65
Fistula		1 (2.5%)	1 (0.9%)	1.0
Bleeding	Grade 2	1 (2.5%)	3 (2.7%)	1.0
	Grade 3	1 (2.5%)	3 (2.7%)	
	Any grade	2 (5%)	6 (5.4%)	0.92
Cardiopathy		1 (2.5%)	5 (4.5%)	0.10
Treatment delay	Number of patients	3 (7.5%)	7 (6.3%)	0.47
	days delay*	85.33 ± 49.92 (30–127)	69.57 ± 121.45 (8–340)	

DDB: double dose bevacizumab; SDB: standard dose bevacizumab; data are n (%); *p*-values were calculated with χ2test

*data are mean ± SD (range) and *p*-value was calculated with *t*-test; all tests were conducted at 0.05 level of significance. Bold the significant *p* value when we have multiple values.

Survival curves were constructed for OS, TTF, PFS1, and PFS2 according to primary tumor location (data not shown) and the corresponding medians and HR were determined. Statistically significant differences were obtained for OS only with a higher median survival for left-sided vs. right-sided tumors: 29 months (IQR: 25–37) vs. 22 months (IQR: 18–30), respectively, (*p* = 0.01).

In the group of patients having received DDB after first progression, no differences between left- and right-sided mCRC were observed in terms of OS, PFS1, PFS2, and TTF.

Patients receiving SDB had significantly improved OS and PFS2 for left-sided mCRC compared to right-sided disease (Table 5). However, TTF and PFS1 were not significantly different between left- and right-sided tumors in patients receiving SDB in second-line treatment.

**TABLE 5:** Characteristics of OS, TTF, and PFS depending on primary tumor location in the DDB and SDB groups.

Item	Left-sided*	Right-sided*	HR (CI 95%)	*p*-value
DDB				
OS	46 (35–56)	27 (21–41)	0.57 (0.26–1.24)	0.15
TTF	31 (22–39)	21 (17–28)	0.60 (0.28–1.27)	0.18
PFS1	16 (14–27)	16 (11–20)	0.73 (0.36–1.49)	0.39
PFS2	9 (7–14)	5 (3–10)	0.48 (0.22–1.03)	0.06
SDB				
OS	27 (24–35)	12 (13–32)	0.54 (0.33–0.88)	**0.01**
TTF	19 (16–23)	15 (11–23)	0.99 (0.41–1.08)	0.10
PFS1	12 (11–14)	10 (7–17)	0.84 (0.53–1.33)	0.45
PFS2	6 (5–8)	4 (3–6)	0.55 (0.33–0.9)	**0.02**

OS: overall survival, TTF: time to treatment failure, PFS1: progression-free survival during first-line treatment, PFS2: progression-free survival during second-line treatment, DDB: double dose bevacizumab, SDB: standard dose bevacizumab; p-values were calculated with log-rank test; HR < 1 means better results in left -sided than in right-sided cases

*all values are displayed as medians (interquartile range) in months. Bold the significant *p* value when we have multiple values.

### First and Second-line Chemotherapy Backbone

We investigated the role of first and second-line chemotherapy partners for bevacizumab to improve our understanding for the impact of chemotherapy in mCRC patients.

When comparing the groups regarding the type of standard chemotherapy regimens (irinotecan vs. oxaliplatin backbone) in first- or second-line treatment, a trend toward a greater OS, TTF, PFS1, and PFS2 was observed for irinotecan-based regimens compared to oxaliplatin-based chemotherapy in both groups, however these differences did not reach statistical significance (Table 6).

**TABLE 6:** Median of OS, TTF, and PFS based on type of chemotherapy in the SDB and DDB groups.

		Irinotecan-based*	Oxaliplatin-based*	*p*-value
DDB	OS	43.5 (30–35)	27 (18–44)	0.06
SDB		25 (22–36)	25 (23–30)	0.50
DDB	TTF	25 (21–36)	24 (17–35)	0.60
SDB		21 (16–30)	16 (15–22)	0.30
DDB	PFS1	16 (14–28)	14 (9–19)	0.07
SDB		13 (12–18)	12 (9–13)	0.30
DDB	PFS2	9 (5–19)	8 (6–12)	0.30
SDB		5 (4–6)	6 (4–9)	0.70

OS: overall survival, TTF: time to treatment failure, PFS1: progression-free survival during first-line treatment, PFS2: progression-free survival during second-line treatment, DDB: double dose bevacizumab, SDB: standard dose bevacizumab; p-values were calculated with log-rank test

*data are median (interquartile range) in months.

### Timing of Bevacizumab Initiation

We investigated the time of initiation of bevacizumab treatment in terms of months of delay of treatment to see whether it explains the differences in OS, TTF, PFS1, and PFS2 between the DDB and SDB groups; for instance, whether PFS1 could be statistically linked to the initiation of bevacizumab. According to guidelines, bevacizumab must be administrated from the first cycle of systemic treatment, without any delays.

PFS1 and TTF were negatively and significantly correlated with delayed bevacizumab initiation in both the DDB and SDB groups of patients (*p* < 0.01). As shown in Table 7, this correlation was moderate.

**TABLE 7:** Correlation between treatment outcomes and delayed initiation of bevacizumab in first-line treatment in DDB and SDB groups.

		Delayed bevacizumabinitiation–in months	
		*r*	*p*-value
OS	DDB	−0.31	0.052
TTF		−0.51	**<0.01**
PFS1		−0.43	**<0.01**
PFS2		−0.39	**0.01**
OS	SDB	−0.19	**0.04**
TTF		−0.41	**<0.01**
PFS1		−0.42	**<0.01**
PFS2		−0.17	0.07

OS: overall survival, TTF: time to tratment failure, PFS1: progression-free survival during first-line treatment, PFS2: progression-free survival during second-line treatment, DDB: double dose bevacizumab, SDB: standard dose bevacizumab; p-value <0.05 indicate statistical significance of Pearson's correlation coefficients (r). Bold the significant p value when we have multiple values.

### Bevacizumab dose Intensity

The analysis of bevacizumab dose intensity revealed significant differences in both first and second-line therapy between the DDB and SDB groups. These differences remained also significant for the overall treatment (first and second-line) (Table 8).

**TABLE 8:** Mean bevacizumab dose intensity in the DDB and SDB patient groups.

	First-line Bev-DI (mg/kg/week)	Second-line Bev-DI (mg/kg/week)	Total Bev-DI (mg/kg/week)
DDB	1.84 ± 0.56 (0.47–3.22)	4.49 ± 2.31 (0.66–12.08)	2.68 ± 1.18 (1.23–7.26)
SDB	1.50 ± 0.64 (0.23–3.06)	2.47 ± 1.05 (0.36–7.74)	1.74 ± 0.52 (0.59–2.97)
*p* value	**0.004**	**<0.001**	**<0.001**

Bev-DI: bevacizumab dose intensity; DDB: double dose bevacizumab, SDB: standard dose bevacizumab; p-values were calculated with *t*-test; data are mean ± SD (range). p value statistical significant.

The Pearson correlation analysis between the total dose of bevacizumab and survival rates is detailed in Table 9.

**TABLE 9:** Correlation between treatment outcomes and total bevacizumab dose.

Outcome variable		Bevacizumab total dose	
		*r*	*p*-value
OS	DDB	0.67	<0.001
TTF		0.77	<0.001
PFS1		0.61	<0.001
PFS2		0.64	<0.001
OS	SDB	0.60	<0.001
TTF		0.80	<0.001
PFS1		0.75	<0.001
PFS2		0.47	<0.001
OS	All	0.59	<0.001
TTF		0.77	<0.001
PFS1		0.68	<0.001
PFS2		0.54	<0.001

OS: overall survival, TTF: time to treatment failure, PFS1: progression-free survival during first-line therapy, PFS2: progression-free survival during second-line therapy, DDB: double dose bevacizumab, SDB: standard dose bevacizumab

*all corresponding p-value are lower than 0.001.

As seen in Table 9, both groups (DDB and SDB) had significantly positive correlations between all the treatment outcomes and the total dose of bevacizumab administered (*p* < 0.001). TTF was positively and strongly correlated with total bevacizumab dose, while OS, PFS1, and PFS2 were moderately correlated with total bevacizumab dose, with a tendency toward strong positive correlation in the case of PFS1 in SDB group (Table 9).

### Subgroup Analysis of Overall Survival

Male gender, age below 65 and an irinotecan-based chemotherapy regimen in first-line were significantly linked to survival advantage among mCRC patients (Table 10).

**TABLE 10:** Subgroup analysis of overall survival hazard ratios.

	N	HR	95% CI	*p*-value
All	151	0.62	0.42–0.91	**0.02**
Gender				
Male	86	0.53	0.31–0.90	**0.02**
Female	65	0.65	0.35–1.18	0.16
Age (years)				
<65	123	0.60	0.39–0.91	**0.02**
≥65	28	0.66	0.22–1.97	0.45
First-line chemotherapy				
Oxaliplatin-based	89	0.87	0.40–1.90	0.73
Irinotecan-based	56	0.49	0.27–0.88	**0.02**
PFS1(months)				
≤12	76	0.58	0.31–1.08	0.09

PFS1: progression-free survival after first-line therapy, HR: hazard ratio; CI: confidence interval; p-values were calculated with log-rank test; A hazard ratio< 1 indicate better treatment in double dose bevacizumab group vs. standard dose bevacizumab group. Bold the significant p value when we have multiple values.

### Was OS Influenced by PFS1 or PFS2?

As shown in Table 11, OS was significantly and positively correlated with each of TTF, PFS1, and PFS2 among all mCRC patients and each of DDB and SDB patient groups (*p* < 0.001). These correlations were moderate to strong according to the Pearson’s correlation analysis.

**TABLE 11:** Correlation between OS and each of PFS and TTF in SDB and DDB groups.

Outcome variable		Overall survival (OS)	
		*r*	*p*-value
PFS1	DDB	0.60	<0.001
PFS2		0.47	<0.001
TTF		0.69	<0.001
PFS1	SDB	0.65	<0.001
PFS2		0.56	<0.001
TTF		0.75	<0.001
PFS1	All	0.65	<0.001
PFS2		0.55	<0.001
TTF		0.75	<0.001

OS: overall survival, TTF: time to treatment failure, PFS1: progression-free survival during first-line therapy, PFS2: progression-free survival during second-line therapy, SDB: standard dose bevacizumab, DDB: double dose bevacizumab.

## Discussion

### Primary Tumor Location

Primary tumor location (PTL) is one of the most important prognostic factors for mCRC. Survival after recurrence was significantly longer in left-sided compared with right-sided colon cancer patients (Cutsem et al., 2016). In first-line treatment with oxaliplatin, fluoropyrimidine and bevacizumab, the laterality of the primary tumor was less important in the maintenance phase of first-line therapy (Jordan et al., 2018). However, PTL remains a significant prognostic factor for second and further lines of treatment (Hegewisch-Becker et al., 2018).

Surprisingly, in our DDB group of patients, no statistically significant differences in terms of OS, TTF, PFS1, and PFS2 in relation with PTL were noted. These results suggest that doubling the dose of bevacizumab overrides the prognostic differences in mCRC according to tumor location. Increasing the dose of bevacizumab could equalize the chance of response to systemic treatment and control of the disease for left-sided and right-sided mCRC patients. However, considering the PTL role in CRC prognosis, Boeckx et al. showed that patients with RAS wild-type left-sided mCRC had a better OS than right-sided disease, regardless of treatment received (Boeckx et al., 2018). In line with this, in a study including 754 patients with first-line therapy of bevacizumab and oxaliplatin backbone regimen, Hegewisch-Becker et al. found significantly better survival in left-sided CRC patients (median survival 24.8 months) compared with right-sided patients (18.4 months), with PTL being the most powerful prognostic factor in multivariate analysis (Hegewisch Becker et al., 2018).

### First and Second-line Chemotherapy Backbone Associated With Bevacizumab

The ESMO guidelines recommend the combination of a standard chemotherapy regimen (irinotecan or oxaliplatin-based) with either anti-VEGF or anti–EGFR therapy in the first and second-line treatment in mCRC patients (Schmoll et al., 2012). The NCCN guidelines recommend bevacizumab in first-line therapy in associations with classical chemotherapy regimens but with modest effect on OS, especially when added to oxaliplatin (Messersmith, 2019).

In our study, both chemotherapy regimens–oxaliplatin or irinotecan backbone were equally effective in combination with bevacizumab, as no significant differences were seen between the DDB and SDB groups. The first-line chemotherapy regimen with oxaliplatin backbone was more frequently used in the SDB group compared with DDB in first-line (*p* < 0.001) while irinotecan was more frequently used in the first-line for DDB patients (*p* = 0.014). Similar with our data, two previous studies conducted by Bendell et al. in ARIES study, on 1,550 patients, and Yamazaki et al. on 395 patients, treated in the first-line with FOLFIRI or FOLFOX combined with bevacizumab, retrieved equivalent OS, PFS or rate of response between arms (Bendell et al., 2012; Yamazaki et al., 2014). Moreover, van Cutsem et al. showed similar efficacy of FOLFOX, FOLFIRI or XELOX combined with bevacizumab in the first-line in the BEAT study on 1914 patients (Van Cutsem et al., 2009). Premature discontinuation of oxaliplatin-based treatment as defined by protocol design altered HR of PFS from 0.63 to 0.83 (Ilic et al., 2016). The main concerns with oxaliplatin-based chemotherapy are its cumulative and slowly reversible neurotoxicity as well as the allergic reactions associated with this regimen (Cetean et al., 2015).

When regimens were compared, the irinotecan-based one seemed to be slightly superior. Ilic et al. and Baraniskin et al. confirmed the advantage of using bevacizumab with chemotherapy (Ilic et al., 2016; Baraniskin et al., 2019).

### Bevacizumab dose Intensity and its use Beyond Progression

In clinical practice, according to the European guideline (ESMO) as well as NCCN guideline, a complete genetic characterization of the tumor (all RAS type) and imaging evaluation are required before choosing a systemic regimen for a particular patient (Cutsem et al., 2016; Messersmith, 2019; Veld et al., 2019). The NCCN guidelines draw attention to the existence of preclinical data suggesting a possible rebound effect after bevacizumab cessation (Veld et al., 2019). Our data revealed lower bevacizumab dose intensity in the first but not in the second-line for SDB. The same effect was also recorded for DDB, with an explicable doubling of the intensity in the second-line. The explanation of these facts resides in the administrative constraints existing in our country. In Romania, for more than a decade the national insurance reimbursed the cost of bevacizumab only after a centralized approval. This fact was responsible for widespread and sometimes prolonged delays in optimal bevacizumab administration in the first-line, which were less encountered in second-line administration. We found no significant difference in first-line delays between the SDB and DDB groups. However, our analysis did show significantly negative correlations between PFS1, TTF, and delayed bevacizumab initiation in both the SDB and DDB groups (*p* < 0.01). In DDB group, PFS2 was moderately and negatively correlated with time of bevacizumab delay, while OS was weakly, yet significantly and negatively correlated with delayed bevacizumab administration in the SDB group. Contrary to first-line administration of bevacizumab in our patients, in the second-line we did not observe significant delays in bevacizumab start of administration. Moreover, the dose of bevacizumab was doubled after disease progression in the DDB group with practical aim to overcome tumor resistance which was translated into a prolonged PFS2 for DDB patients compared to SDB. Whether first-line delay of bevacizumab administration could influence PFS2–it must be interpreted with caution, due to the retrospective nature of our analysis with possible bias in patient recruitment and inclusion process. Differences in dose intensity in the first-line treatment may raise questions regarding an excess of bevacizumab or classic chemotherapy partner toxicity in the first-line for the SDB group.

One of the first clinical trials to address the question whether bevacizumab should be continued after disease progression was the ML 18147 phase III trial (Bennouna et al., 2013). The trial was positive in terms of OS, considering the same dose of bevacizumab as in the first-line (2.5 mg/kg body/week). Moreover, in the BRiTE study, Grothey et al. defined “bevacizumab beyond progression” (BYP) differently referring to patients who continued treatment after disease progression within two months at maximum. Median OS and survival after progression were better in patients who received bevacizumab after first progression (19.2 vs. 9.5 months) (Grothey et al., 2014). In the ARIES study on 1,550 patients, Bendell et al. reported that a cumulative dose of bevacizumab represented a significant prognostic factor for survival after first progression (Bendell et al., 2012). The same results were retrieved by Cartwright et al. on 573 patients as determined by improved OS (27.9 vs. 14.6 months) and post-progression survival (21.4 vs. 10.1 months) (Cartwright et al., 2012).

In the same context, in a phase III trial on 185 patients treated with second-line bevacizumab with or without FOLFIRI or FOLFOX (depending on the first-line treatment already administered), Masi et al. found a statistically significant PFS and OS advantage for bevacizumab continuation or reintroduction in the second-line over chemotherapy alone in all the analyzed subgroups (Masi et al., 2015).

### Toxicity of Bevacizumab in mCRC Patients

Bevacizumab has some specific adverse effects like hypertension, proteinuria, gastro-intestinal perforation, thrombosis, pulmonary thromboembolism and hemorrhage (Feliu et al., 2015; Dionísio de Sousa et al., 2016).

In our analysis, no statistically significant differences were found in the number of hypertensive cases between the SDB and DDB groups, but in the DDB group grade 3 hypertension was twice as frequent as in the SDB group (*p* = 0.04). Hypertension (grade 3 and 4) is a common adverse event to bevacizumab treatment with an incidence of 0.4 and 17.9% (Dionísio de Sousa et al., 2016). In a small retrospective analysis of 79 patients, grade 2 and 3 of hypertension during bevacizumab administration was predictive of PFS but not OS improvement (Feliu et al., 2015). Our data also revealed that proteinuria was more frequent in the DDB than in the SDB group, reaching statistical significance. Proteinuria was reported in 0.7–54.7% of patients (Feliu et al., 2015). Proteinuria was not correlated with OS or PFS advantage in a phase II trial (Lee et al., 2019). Both hypertension and proteinuria were statistically correlated with the duration of bevacizumab administration, higher doses of bevacizumab may increase the risk, due to a cumulative effect (Loupakis et al., 2018). As for the other bevacizumab adverse events, no significant differences in bleeding, perforation, cardiomyopathy, thrombosis and emboli were found between groups. Treatment was not delayed in the DDB group despite the higher dose in the second-line since no untreatable toxicity occurred. However, perforations were reported in 0.2–1% of patients, bleeding in 10–20% and thrombosis and thromboembolism events in 2.8–17.3% of patients (Feliu et al., 2015).

### Subgroup Analysis of Overall Survival

Older age could be a factor for under-treatment reported by Raab et al. (Raab et al., 2019), despite the fact that bevacizumab could improve OS and PFS for this category of patients, as showed by Pinto et al. (Pinto et al., 2017).

In our study, male gender, age below 65 and irinotecan-based chemotherapy in the first-line were significantly linked to a survival advantage. No PFS1 cut-off value was found to be associated with better OS. In the same line, the results of Loupakis et al. that retrospectively analyzed the results of NO 16966 and AVF2107g phase III trials, including patients with mCRC treated in the first-line with bevacizumab and standard chemotherapy showed no OS advantage in any subcategory (Loupakis et al., 2018).

In a pooled analysis of 2,879 patients included in major published trials (COIN, OPUS, AGITG, CRYSTAL, FOCUS 2, and COIN-B), Salem et al. showed that left-sided colon cancer patients were mostly elderly females (over the age of 70 years) (Salem et al., 2018). However, considering the Rahman et al. data, gender was not shown to be predictive for OS (Abdel-Rahman, 2019).

### Is OS Influenced by PFS1 or PFS2?

Post-progression survival is a very important factor that may characterize better clinical outcomes after first disease progression. Petrelli et al. showed that in 16,408 patients included in 34 phase III randomized clinical trials, a good correlation between OS and post-progression survival (PFS2) was observed (Petrelli and Barni, 2013).

In our study,PFS1, PFS2, TTF, and OS were all higher in the group of mCRC patients treated with DDB after disease progression (Table 3) (Bochis et al., 2020). In previously published data PFS1 was shown to have a strong influence on OS (Petrelli and Barni, 2013). However, in our study, a positive moderate correlation between PFS1, PFS2, and OS with total bevacizumab dose was found in both groups of patients. PFS1 in SDB group showed a tendency to strong positive correlation. All Pearson’s correlations examined were statistically significant (*p* < 0.001). PFS1 should not have been dependent on bevacizumab dose intensity since the same first-line doses were administered in both the DDB and SDB groups. The retrospective nature of our study may explain this finding. An analysis of 22,736 patients included in 50 clinical trials found a good correlation between PFS and OS in chemotherapy regimens but less so for trials on monoclonal antibodies (Petrelli and Barni, 2013). Within 20,438 patients, Sidhu et al. observed a weak correlation between the response rate (RR) and OS, lower than the correlation coefficient for PFS and OS (Sidhu et al., 2013).

The limitations of our study may reside in several factors such as the lack of patient randomization at inclusion and variations in the induction treatment and should be considered as a potential source of bias. The unbalanced use of an oxaliplatin backbone regimen in first and second-line therapy in both the SDB and DDB groups may be a factor influencing the general results of our study, due to well-known differences in terms of rate of response, type, and intensity of toxicity compared with irinotecan. The strength of our analysis resides in the data extracted from the medical records of unselected patients with mCRC treated with systemic treatment. The inclusion and exclusion criteria established in this study were meant to create a real-life situation that medical oncologists may be faced with. By avoiding the over-selection of patients imposed by the constraints of a clinical trial, we wished to have a better chance of determining the advantage (if any) of doubling the bevacizumab dose in mCRC patients after first disease progression. In the EMA market-approval for bevacizumab in mCRC, both treatment strategies–standard and double dose–are mentioned, without any recommendation on when to use the double dose. The scarce clinical data available need to be completed with the experience of cancer centers, regional databases, and randomized phase III trials to provide the best proof of efficacy of one of the two strategies. No definitive conclusion can be drawn according to the available data.

## Conclusion

Our data demonstrated that doubling the dose of bevacizumab after first progression may improve OS, PFS1, PFS2, and TTF. Moreover, an increasing dose of bevacizumab may lead to better outcomes in the DDB group. However, doubling the dose of bevacizumab was not associated with increased toxicities except for grade 3 hypertension, which was manageable, without negative influence on treatment administration. Irinotecan-based chemotherapy regimens in the first-line significantly could be a preferential partner for bevacizumab in mCRC patients.

## Data Availability

The datasets generated for this study will not be made publicly available The datasets for this article are not publicly available because: GPDR legislation. Requests to access the datasets should be directed to CC, (calincainap2015@gmail.com).
